# 再生障碍性贫血患者HLA-A抗原表达缺失的研究

**DOI:** 10.3760/cma.j.issn.0253-2727.2021.11.012

**Published:** 2021-11

**Authors:** 会勤 张, 化泉 王, 薇薇 齐, 春燕 刘, 莉民 邢, 蓉 付, 宗鸿 邵

**Affiliations:** 1 天津医科大学总医院血液科 300052 Department of Hematology, General Hospital, Tianjin Medical University, Tianjin 300052, China; 2 天津医科大学第二医院血液科 300211 Department of Hematology, The Second Hospital of Tianjin Medical University, Tianjin 300211, China

再生障碍性贫血（AA）是一种以全血细胞减少为表现的骨髓造血功能衰竭综合征，主要临床表现是出血、贫血和感染等[1]。AA的发病机制尚未完全明确。细胞毒性T淋巴细胞（CTL）亢进导致的造血干/祖细胞（HPSC）免疫损伤是主要发病环节。宿主的遗传易感性是免疫损伤的基础[2]。CTL通过人类白细胞抗原（HLA）-Ⅰ类分子识别HPSC自身抗原，激发自身免疫反应机制，在引发骨髓造血功能衰竭的自身免疫反应中发挥重要作用[3]–[4]。本文旨在研究AA患者外周血HLA-A等位基因在多谱系上的表达是否缺失，及对免疫抑制治疗（IST）疗效的影响，分析HLA-A的表达与临床指标的相关性。

## 病例与方法

1. 病例：回顾性分析2017年4月至2018年3月天津医科大学总医院血液内科收治的AA患者36例，其中初诊未治疗组患者17例，男8例，女9例，中位年龄39（17～73）岁。治疗组［经抗胸腺/淋巴细胞球蛋白（ATG/ALG）、环孢素A（CsA）等治疗后］患者19例，男10例，女9例，中位年龄41（13～68）岁。依据《再生障碍性贫血诊断与治疗中国专家共识（2017年版）》[5]来进行诊断、治疗和疗效评价，疗效评价时间是IST后6个月。患者信息详见表1。本研究方案经天津医科大学总医院伦理委员会批准，所有研究对象或其监护人均知情同意并签署知情同意书。

**表1:** 36例再生障碍性贫血（AA）患者的基本临床特征

临床特征	初诊未治疗组（17例）	治疗组（19例）
性别（例，男/女）	8/9	10/9
中位年龄［岁，*M*（范围）］	39（17~73）	41（13~68）
血常规		
中位HGB［g/L，*M*（范围）］	61（42~86）	98（56~148）
中位PLT［×10^9^/L，*M*（范围）］	9（1~37）	51（9~293）
中位ANC［×10^9^/L，*M*（范围）］	0.37（0.16~1.97）	2.94（1.50~8.54）
中位Ret［×10^9^/L，*M*（范围）］	13.0（4.4~56.1）	41.5（5.2~84.9）
IST前疾病严重程度（例）		
VSAA	2	4
SAA（除外VSAA）	9	10
NSAA	6	5
研究距诊断的时间［月，*M*（范围）］	3（1~7）	11（3~42）
研究距IST的时间［月，*M*（范围）］	NA	6（2~24）
输血依赖（例）		
红细胞输注依赖	16	2
血小板输注依赖	13	1
感染（例）		
呼吸道感染	5	1
皮肤感染	1	0
口腔感染	1	1
IST方案（例）^a^		
ATG/ALG+CsA	9	10
CsA	8	9

注：ANC：中性粒细胞绝对计数；Ret：网织红细胞绝对计数；VSAA；极重型再生障碍性贫血；SAA：重型再生障碍性贫血；NSAA：非重型再生障碍性贫血；IST：免疫抑制治疗；ATG/ALG：抗胸腺/淋巴细胞球蛋白；CsA：环孢素A；NA：不适用。^a^初诊未治疗组完成HLA-A2和A9检测后接受IST方案治疗

2. HLA-A2和A9检测：取3 ml外周血，肝素抗凝；各取50 µl外周血，加入到空白对照管（编号1）、同型对照管（编号02和03）、实验管（编号2和3）中，PE-HLA-A2和A9抗体分别加入实验2和3号管中，IgG1-PE分别加入同型对照02和03管中，空白对照管中不加PE抗体。试管编号2、02中加入Percp-CD3、APC-CD19、FITC-CD56；试管编号3、03中加入APC-CD33、FITC-CD15，震荡摇匀，避光孵育15 min后每管加入1 ml溶血素，混匀，避光孵育10 min，427×*g*离心5 min；离心后弃上清，每管加入1 ml PBS，427×*g*离心5 min；离心后弃上清，每管加入100 µl PBS；上流式细胞仪进行检测，每个样品收集30 000个细胞。

3. 阵发性睡眠性血红蛋白尿症（PNH）克隆检测：取3 ml外周血，肝素抗凝。采用CD59检测红细胞及荧光标记的嗜水气单胞菌溶素变异体（FLAER）联合CD24或CD14检测粒细胞和单核细胞的PNH克隆比例。

4. 统计学处理：应用SPSS21.0统计软件进行分析，计量资料数据用均数±标准差（*x±s*）表示，两组间比较采用独立样本*t*检验（数据符合正态分布）或Wilcoxon秩和检验（数据不符合正态分布），应用Spearman相关性分析描述指标间线性关联；分类变量组间比较采用Fisher精确概率法。*P*<0.05为差异有统计学意义。

## 结果

1. AA患者外周血白细胞上HLA-A的表达：17例初诊未治疗组患者中6例（35.29％）出现HLA-A2部分表达缺失，男4例，女2例，缺失细胞比例为19.47％（7.42％～60.50％）；1例（5.89％）女性出现HLA-A9部分表达缺失，缺失细胞比例为51.74％。19例治疗组患者中5例（26.32％）出现HLA-A2部分表达缺失，男2例，女3例，缺失细胞比例为27.01％（5.69％～71.83％）；3例（15.79％）HLA-A9 部分表达缺失，均为男性，缺失细胞比例分别为49.31％、90.26％和82.17％。

2. 不同白细胞亚型HLA-A表达情况：11例检测到HLA-A2部分表达缺失的AA患者中，11例都存在中性粒细胞（CD15^+^）HLA-A2部分表达缺失，10例检测到单核细胞（CD33^+^）HLA-A2部分表达缺失，8例检测到NK细胞（CD56^+^）HLA-A2部分表达缺失，3例检测到B淋巴细胞（CD19^+^）HLA-A2部分表达缺失，2例检测到T淋巴细胞（CD3^+^）HLA-A2部分表达缺失。

4例检测到HLA-A9部分表达缺失的AA患者中，4例均存在中性粒细胞（CD15^+^）与单核细胞（CD33^+^）HLA-A9部分表达缺失，2例检测到NK细胞（CD56^+^）HLA-A9部分表达缺失，1例检测到B淋巴细胞（CD19^+^）HLA-A9部分表达缺失，未检测到T淋巴细胞（CD3^+^）HLA-A9部分表达缺失。

3. HLA-A部分表达缺失与PNH克隆的关系：15例存在HLA-A部分表达缺失的AA患者中3例（20.0％）PNH克隆阳性，21例不存在HLA-A部分表达缺失的AA患者中8例（38.1％）PNH克隆阳性（*P*＝0.295）。

4. HLA-A表达缺失与IST疗效关系：初诊未治疗组中，7例HLA-A表达缺失患者中5例（71.4％）IST有效；10例HLA-A表达无缺失患者中4例（40.0％）IST有效。治疗组中，8例HLA-A表达缺失患者中5例（62.5％）IST有效；11例HLA-A表达无缺失患者中5例（45.5％）有效。

5. HLA-A等位基因的表达缺失与临床指标的相关性：HLA-A2等位基因表达缺失比例与中性粒细胞绝对计数呈负相关（*r*_s_＝−0.645，*P*＝0.032），与HGB呈正相关（*r*_s_＝0.691，*P*＝0.019），与WBC、PLT和网织红细胞绝对计数（Ret）均无明显线性相关性（*P*值均>0.05）。HLA-A9等位基因表达缺失比例与WBC、HGB、PLT和Ret等均无明显线性相关性（*P*值均>0.05）。

## 讨论

CTL异常活化及其功能亢进是AA的主要发病机制。CTL通过HLA-Ⅰ类分子识别和杀伤HPSC，从而导致骨髓衰竭。Katagiri等[6]发现部分AA患者6号染色体短臂杂合子缺失，致使HLA等位基因表达缺失，从而导致部分HSPC发生免疫逃逸，避免被CTL杀伤。

我们通过检测HLA-A2和A9的表达，发现部分AA患者存在HLA-A等位基因的表达缺失。HLA可能参与AA的发病机制，其表达的缺失使HSPC发生免疫逃逸，避免被杀伤。我们将AA患者分为初诊未治疗组与已行IST治疗组，发现初诊未治疗组的HLA-A的表达缺失率为41.1％，高于Katagiri等[2]报道的结果。这可能因为两者应用的检测技术不同，即FCM检测缺失率高于SNP检测。另外，部分AA患者HLA-A的表达缺失不是6号染色体短臂杂合子缺失导致，而是HLA-A等位基因的编码区序列的突变，或者其他体细胞基因突变导致的[7]–[9]。

Maruyama等[10]发现部分AA患者存在HLA-A的表达缺失，表达缺失可出现在白细胞上的多谱系中（中性粒细胞、单核细胞、B淋巴细胞和T淋巴细胞）。但并不是所有患者在白细胞多谱系上均有表达缺失，部分患者在两系或者三系上表达缺失，部分患者仅在一系上表达缺失。中性粒细胞和单核细胞上表达缺失的AA患者相对较多，淋巴细胞上的表达缺失的相对较低。我们也发现AA患者在白细胞多谱系上存在HLA-A表达缺失，可以累及一系或者多系，各系的表达缺失率有差异。我们研究发现，NK细胞也存在HLA-A的表达缺失，缺失率稍低于中性粒细胞和单核细胞，但高于B淋巴细胞和T淋巴细胞。

我们研究亦发现，HLA-A表达缺失患者PNH克隆阳性比例低于HLA-A表达无缺失患者。这可能是AA HPSC逃避T细胞免疫攻击时，会选择HLA表达缺失的方式，也可以选择PNH克隆缺失的方式。部分患者同时存在这两种缺失方式[11]。

有研究报道初诊未治疗组HLA表达缺失AA患者IST的有效率高达100％，显著高于HLA表达无缺失患者[10]。本研究中，初诊未治疗组HLA-A表达缺失患者IST有效率（71.4％）高于表达无缺失患者（40.0％），但并未达到100.0％，治疗组HLA-A表达缺失患者IST有效率（62.5％）也高于表达无缺失患者（45.5％），由于病例数较低未进行统计学比较。未来需要大样本进一步探索HLA表达缺失与IST疗效是否在统计学水平具有显著性差异。

我们发现HLA-A2表达缺失与ANC呈负相关，与HGB呈正相关性。提示在AA患者中，HLA-A2表达缺失的HPSC可能偏向红系分化，逃避免疫损伤；但HLA-A2表达缺失的中性粒细胞没有逃避免疫杀伤。不同HLA表达缺失后的HPSC的增殖分化功能可能不同，有待于进一步研究。
